# The Effect of Simulating Different Intermediate Host Snail Species on the Link between Water Temperature and Schistosomiasis Risk

**DOI:** 10.1371/journal.pone.0087892

**Published:** 2014-07-02

**Authors:** Nicky McCreesh, Mark Booth

**Affiliations:** School of Medicine, Pharmacy and Health, Durham University, Durham, United Kingdom; University of Minnesota, United States of America

## Abstract

**Introduction:**

A number of studies have attempted to predict the effects of climate change on schistosomiasis risk. The importance of considering different species of intermediate host snails separately has never previously been explored.

**Methods:**

An agent-based model of water temperature and *Biomphalaria pfeifferi* population dynamics and *Schistosoma mansoni* transmission was parameterised to two additional species of snail: *B. glabrata* and *B. alexandrina*.

**Results:**

Simulated *B. alexandrina* populations had lower minimum and maximum temperatures for survival than *B. pfeifferi* populations (12.5–29.5°C vs. 14.0–31.5°C). *B. glabrata* populations survived over a smaller range of temperatures than either *B. pfeifferi* or *B. alexandrina* (17.0°C–29.5°C). Infection risk peaked at 16.5°C, 25.0°C and 19.0°C respectively when *B. pfeifferi*, *B. glabrata* and *B. alexandrina* were simulated. For all species, infection risk increased sharply once a minimum temperature was reached.

**Conclusions:**

The results from all three species suggest that infection risk may increase dramatically with small increases in temperature in areas at or near the currents limits of schistosome transmission. The effect of small increases in temperature in areas where schistosomiasis is currently found will depend both on current temperatures and on the species of snail acting as intermediate host(s) in the area. In most areas where *B. pfeifferi* is the host, infection risk is likely to decrease. In cooler areas where *B. glabrata* is the host, infection risk may increase slightly. In cooler areas where *B. alexandrina* is the host, infection risk may more than double with only 2°C increase in temperature. Our results show that it is crucial to consider the species of intermediate host when attempting to predict the effects of climate change on schistosomiasis.

## Introduction

Little is known about likely effects of climate change on schistosomiasis transmission[1], however neglecting the issue may have a negative effect on control programs and elimination goals by concentrating resources in the wrong areas. Some empirical studies suggest that increasing temperatures may already be expanding the range of schistosomiasis in Uganda, allowing transmission to occur at increasing altitudes[2]–[4]. Control programs that do not adapt to the changing distribution of schistosomiasis, and changing intensities of infection in different areas, are unlikely to make efficient use of the limited resources available to them.

Different species of *Biomphalaria* and *Bulinus*, the intermediate hosts of *Schistosoma mansoni* and *S. haematobium* respectively, have very different distributions[5], habitats[6], and temperature requirements[7]. Despite this, previous attempts to develop models of schistosomiasis and temperature and/or predict the effects of climate change on schistosomiasis have largely ignored the issue of different intermediate host species. One model of *S. japonicum* transmission and climate change in China used data from a single host species only (*Oncomelania hupensis*)[8], however most previous models of *S. mansoni* and *S. haematobium* have been parameterised using data from multiple species, or even genera, of snail[9]–[12]. To date, the only model parameterised to a single species of *Biomphalaria* or *Bulinus* snail is an agent-based model of water temperature and *Biomphalaria pfeifferi* population dynamics and *S. mansoni* transmission[13].

In this paper, we modify our previously published *B. pfeifferi* model[13] to allow us to simulate two additional species of *S. mansoni* intermediate host snails: *Biomphalaria glabrata* and *Biomphalaria alexandrina*. These species were selected due to the very limited amount of empirical data available on other *Biomphalaria* species[6]. *B. pfeifferi* is the most widespread intermediate host of *S. mansoni* in Africa[5], and can be found in a range of different types of water body including streams[14], lakes[15], reservoirs[16], irrigated areas[17]–[19], and rice-paddies[20]. *B. alexandrina* is found in North Africa only, in Egypt, north Sudan, and north-west Libya[5], [21]. It is very common in the water supply and drainage networks of the Nile Delta[21], [22], and can also be found in springs, streams and the edges of swamps[21]. *B. glabrata* has a widespread distribution in South America and the Caribbean[23], and can be found in pools, marshes, and streams[24].

This study is the first to explore how the population ecology of different species of snail may respond to increasing temperatures, and how this could impact on future schistosomiasis infection risk. It addresses a crucial issue that has been largely ignored in previous modelling efforts, but which nonetheless sits at the forefront of issues to be investigated if adaptation to climate change is to be achieved.

## Methods

### Model description

**Figure 1:**
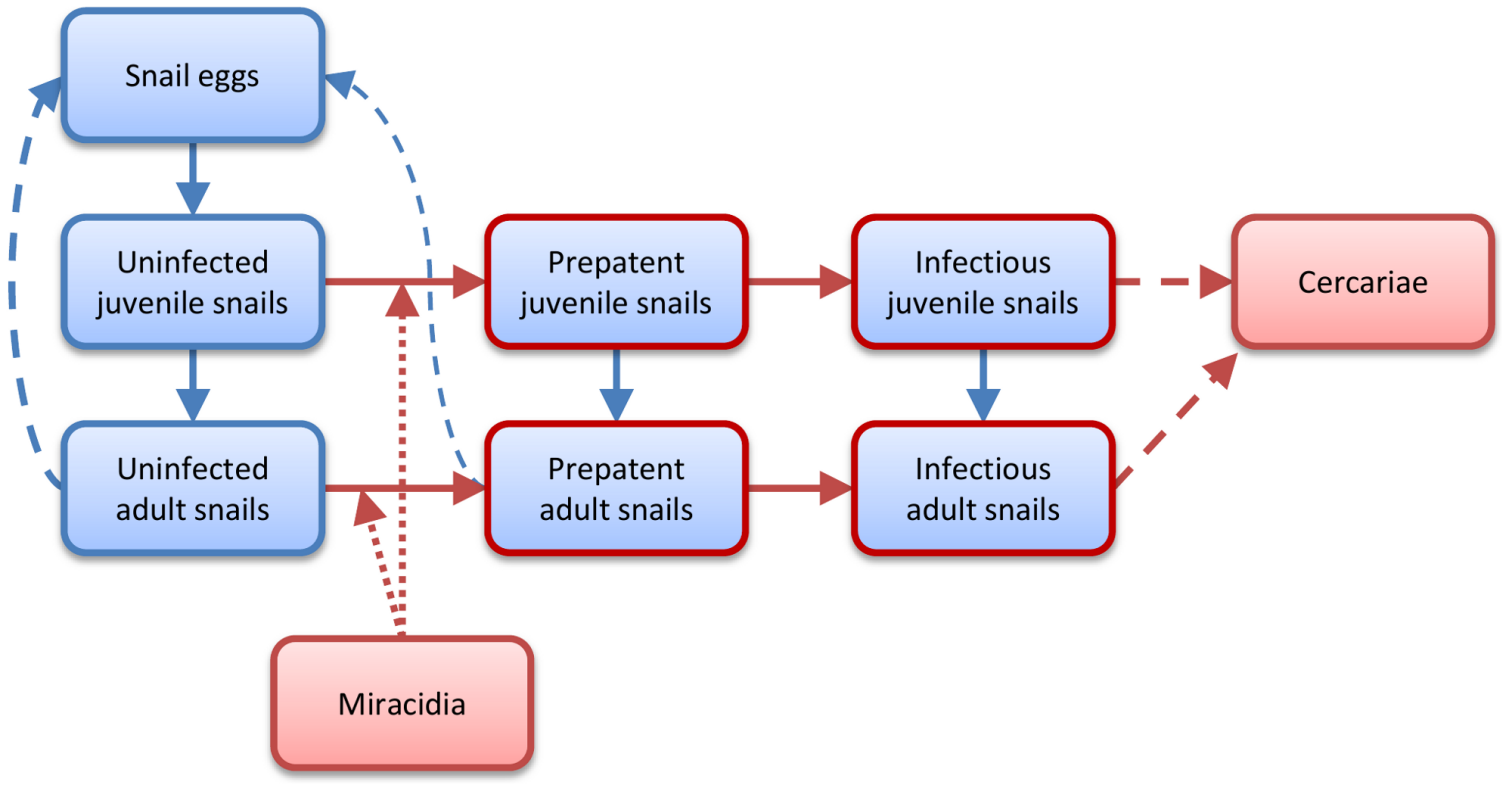
Diagram of the model structure. Boxes show types of agents. Solid arrows show where agents can change from one type into another. Dashed lines show the production of one type of agent by another. Dotted lines indicate infection. Red outlines and arrows indicate the presence of schistosomes. Agents of all types can die and be removed from the model.

The model used is described in full in McCreesh and Booth[13]; a brief description is provided here. The model is an agent-based model of snail population dynamics and infection with schistosomes. All temperature sensitive stages of the snail and schistosome lifecycles are represented by agents (snail eggs, juveniles and adults, miracidia, and cercariae), and the model has a time step of one hour. Figure 1 shows a diagram of the model structure. Snails are born into the model as eggs, and develop at a temperature- dependent rate. When snail egg development is complete, they hatch into juvenile snails. Juvenile snails, in turn, develop at a temperature-dependent rate, becoming adult snails when development is complete. Adult snails produce snail eggs at a rate which is dependent on temperature and the number of snails in the model (to account for reduced fecundity at high snail densities). Snail eggs, juveniles and adults die and are removed from the model at a rate which is dependent on temperature and the number of snails in the model.

Miracidia are introduced into the model at a constant rate. They gain biological age at a temperature-dependent rate and die at a rate that depends on their biological age. They infect juvenile and adult snails at a rate that depends on their biological age and the water temperature. Once infected, juvenile and adult snails become prepatent. Their schistosome infections develop at a temperature-dependent rate, and once development is complete the snails become infectious. Adult snails cease to produce eggs halfway through schistosome development, and infectious snails have increased mortality rates. Once infectious, juvenile and adult snails produce cercariae at a rate which depends on the temperature and the hour of the day. Cercariae gain biological age at a temperature-dependent rate, and die at a biological age dependent rate.

The main output of the model is ‘infection risk’. This is equal to the number of cercariae in the model, adjusted for their decreasing infectiousness with increasing biological age. Humans and adult worms are not simulated, and the rate of miracidia introduction is not linked to the number of cercariae or infection risk. This is because the ‘correct’ linking function would depend on localised factors such as sanitation practices, water contact behaviour, and immunity. Not simulating this link allows the findings of the model to be applied to any area where the relevant intermediate host snail species are found. This issue is discussed in detail in McCreesh and Booth[13].

### Model parameterisation

**Figure 2:**
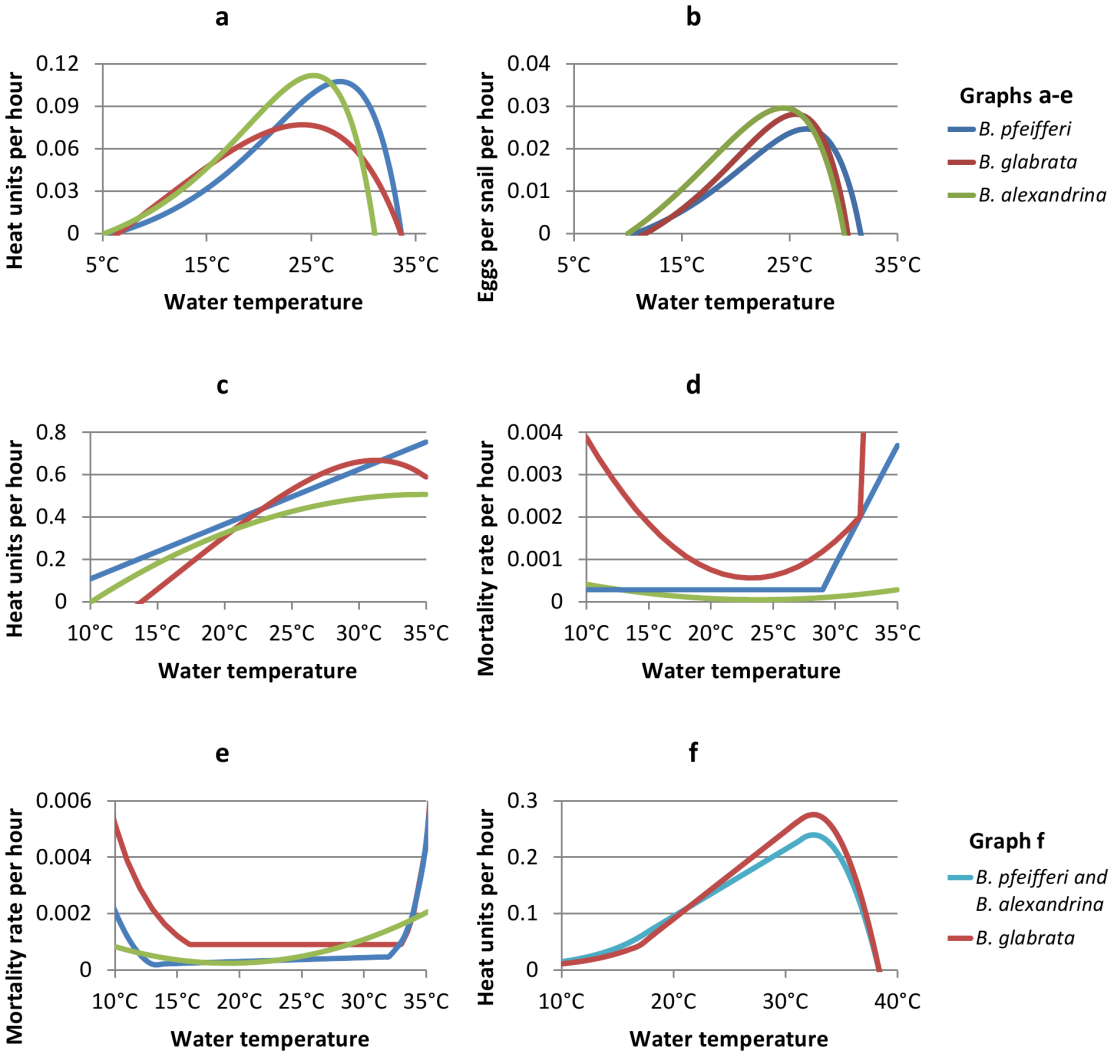
Comparison of parameters used in models of *B. pfeifferi*, *B. glabrata* and *B. alexandrina*. a) Juvenile development. Heat units gained per hour. Snails become adults and start producing eggs once they have gained 100 heat units. b) Egg production. Eggs per snail per hour. c) Egg development. Heat units gained per hour. Egg hatch once they have gained 100 heat units. d) Egg mortality rate per hour. e) Uninfected and prepatent snail mortality rate per hour. f) Parasite development within the snail. Heat units gained per hour. Snails become infectious once they have gained 100 heat units. Blue lines show parameters for *B. pfeifferi*, red for *B. glabrata*, green for *B. alexandrina*, and turquoise for both *B. pfeifferi* and *B. alexandrina*.

The model was parameterised separately for three different species of *Biomphalaria*: *B. pfeifferi*, *B. glabrata* and *B. alexandrina*, using data from a range of different laboratory experiments and field studies. Details of the model parameterisation to *B. pfeifferi* are given in McCreesh and Booth[13], and details of the model parameterisation to *B. glabrata* and *B. alexandrina* are given in Figures S1–S6, and Tables S1–S3 in Model Parameterisation S1. Figure 2 illustrates values taken by the parameters which vary between snail species in the model: a) the juvenile development rate, b) the egg production rate, c) the egg development rate, d) the egg mortality rate, e) the uninfected and prepatent snail mortality rates, and f) the schistosome development rate.

### Experiments

The model was run separately for *B. pfeifferi*, *B. glabrata* and *B. alexandrina* at all water temperatures at which the simulated snail populations could survive indefinitely, with temperature increasing in 0.5°C increments. Outputs were averaged over a minimum of one year and 200 runs. Outputs were averaged over larger numbers of longer runs when necessary to reduce stochasticity. The number of snails in the model was calculated as the total number of uninfected, prepatent and infectious juvenile and adult snails.

In the models for all three snail species, mortality rates estimated from laboratory data were multiplied by 1.35 to account for increased mortality in natural conditions (see Model Parameterisation S1 for details). Estimates of mortality rates in wild snail populations suggest that mortality rates can be much higher than this in some circumstances however[22], [24], [25]. The effect of this was explored by further doubling mortality rates for all snails (juvenile and adult; uninfected, prepatent and infectious).

A number of intermediate results were also calculated by temperature for each snail species. These results were calculated from the equations used in the model only, and were designed to help improve understanding of the overall model results. They were:

1. The mean proportion of eggs that hatch, calculated from egg development rates and egg mortality rates
2. The mean proportion of juvenile snails that survive to become adults, calculated from juvenile development rates and the uninfected/prepatent mortality rates.
3. The mean proportion of infected snails that survive to become infectious, calculated from parasite development rates and the prepatent mortality rates.
4. The median lifetime cercaria production of infectious snails, calculated from the cercariae production rates and the infectious snail mortality rates.

As these intermediate results were calculated from the input parameters only, and not from model output, 2) to 4) assume that there is no density-dependent increase in mortality rates. 2) also assumes that no juvenile snails develop patent infections before becoming adults (which would increase their mortality rate).

## Results

### Snail population dynamics

**Figure 3:**
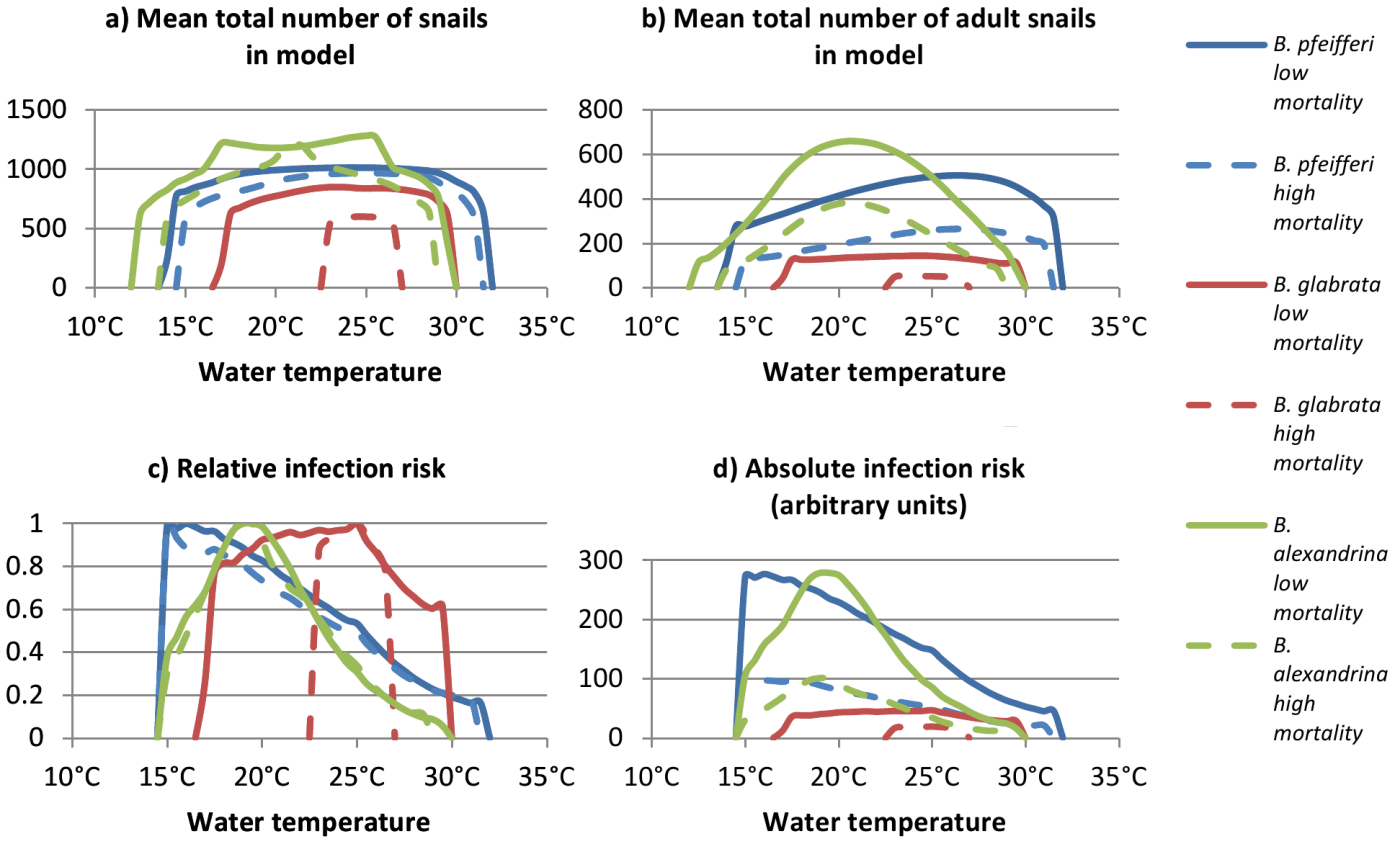
Number of snails and relative infection risk, with high and low mortality rates. a) Mean total number of snails in the model. b) Mean total number of adult snails in the model. c) Infection risk, relative to the maximum risk for the same snail species with the same mortality rates. d) Absolute infection risk (arbitrary units). Blue lines show *B. pfeifferi*, red lines show *B. glabrata* and green lines show *B. alexandrina.* Solid lines show results with a low mortality rate and dashed lines with a high mortality rate.

When the lower mortality rates were simulated, the mean total number of *B. glabrata* in the model was lower than the mean total number of *B. pfeifferi* at all water temperatures, and lower than the number of *B. alexandrina* at all temperatures below 29.5°C (figure 3a). The mean total number of *B. alexandrina* was higher than the number of *B. pfeifferi* at all temperatures below 27.0°C. The maximum mean total number of *B. pfeifferi*, *B. glabrata* and *B. alexandrina* were 1015, 850 and 1284 respectively, at 24.5°C, 23.5°C and 25.0°C. Snail numbers were above 90% of their maximum between 17.0°C and 29.5°C for *B. pfeifferi*, 20.0°C and 28.5°C for *B. glabrata*, and 17.0°C and 25.5°C for *B. alexandrina.* Differences were greater between the maximum mean total number of adult snails in each low mortality rate model, with the *B. pfeifferi* model peaking at 506 adult snails at 26.5°C, the *B. glabrata* model at 145 snails at 24°C, and the *B. alexandrina* model at 661 snails at 20.5°C (figure 3b).

Simulating the higher mortality rates reduced both mean snail and adult snail numbers at all temperatures for all snail species, with the exception of the mean number of *B. alexandrina* at 20.5°C to 21.5°C, where snail numbers were 0.09% to 2.03% higher in the higher mortality scenario. This occurred as a result of the density dependence functions used in the model, and would not necessarily occur in wild snail populations. The presence and magnitude of the dip depends on the density functions used. Reductions in the mean number of snails, over the range of temperatures at which the simulated snail populations could survive indefinitely, varied between 4% and 28% for *B. pfeifferi*, 29% and 36% for *B. glabrata*, and 8% and 28% for *B. alexandrina*. Reductions in the mean number of adult snails ranged between 47% and 56% for *B. pfeifferi*, 63% and 66% for *B. glabrata*, and 42% and 59% for *B. alexandrina*.

The range of temperatures between which simulated snail populations could survive indefinitely was smallest for *B. glabrata*, with snail populations unable to survive at temperatures outside 17.0°C to 29.5°C, compared to 14.0°C to 31.5°C for *B. pfeifferi*. Simulated *B. alexandrina* populations survived at lower temperatures than either *B. glabrata* or *B. pfeifferi*, with a minimum temperature for survival of 12.5°C, but did not survive as well as *B. pfeifferi* at high temperatures, dying out at temperatures above 29.5°C. Doubling snail mortality rates slightly reduced the range of temperatures at which simulated *B. pfeifferi* and *B. alexandrina* populations could survive indefinitely to 15.0°C to 31.0°C and 14°C to 28.5°C respectively. Doubling mortality rates had a much greater effect on simulated *B. glabrata* populations, reducing the range of temperatures at which they could survive indefinitely to only 23.0 to 26.5°C.

### Infection risk

With the lower mortality rates, infection risk was highest at 16.5°C when *B. pfeifferi* was simulated, at 19.0°C when *B. alexandrina* was simulated, and at 25.0°C when *B. glabrata* was simulated (figure 3c). Either side of these temperatures infection risk fell when simulating all snail species, however infection risk remained high over a wide range of temperatures when *B. glabrata* was the intermediate host snail species. Infection risk remained above 80% of its maximum value at all temperatures between 18°C and 26.5°C in the *B. glabrata* model, between 15°C and 20.5°C in the *B. pfeifferi* model, and between 18°C and 21°C in the *B. alexandrina* model. There was a risk of infection at all temperatures at which simulated *B. glabrata* populations could survive indefinitely. There was no infection risk below 15.0°C in any model, while simulated *B. pfeifferi* and *B. alexandrina* populations could survive indefinitely at temperatures as low as 14.0°C and 12.5°C respectively.

Absolute infection risk was highest when *B. pfeifferi* was simulated at all temperatures except between 18.5°C and 22.0°C, when absolute infection risk was highest when *B. alexandrina* was simulated (Figure 3d). Absolute infection risk was lowest when *B. glabrata* was simulated at all temperatures below 28.5°C. Above this temperature, absolute infection risk was lowest when *B. alexandrina* was simulated. Compared to when *B. pfeifferi* was simulated, maximum absolute infection risk was 0.6% higher when *B. alexandrina* was simulated and 83% lower when *B. glabrata* was simulated.

Doubling snail mortality effects had little effect on the relationship between water temperature and infection risk (relative to maximum infection risk in the same scenario), beyond eliminating infection risk at temperatures at which the snail populations were unable to survive indefinitely. It did however reduce absolute infection risk by 53–65% for *B. pfeifferi*, 58–71% for *B. alexandrina* and 58–63% for *B. glabrata* (within the range of temperatures that simulated snail populations were able to survive indefinitely).

### Snail survival by stage, and median cercariae production

**Figure 4:**
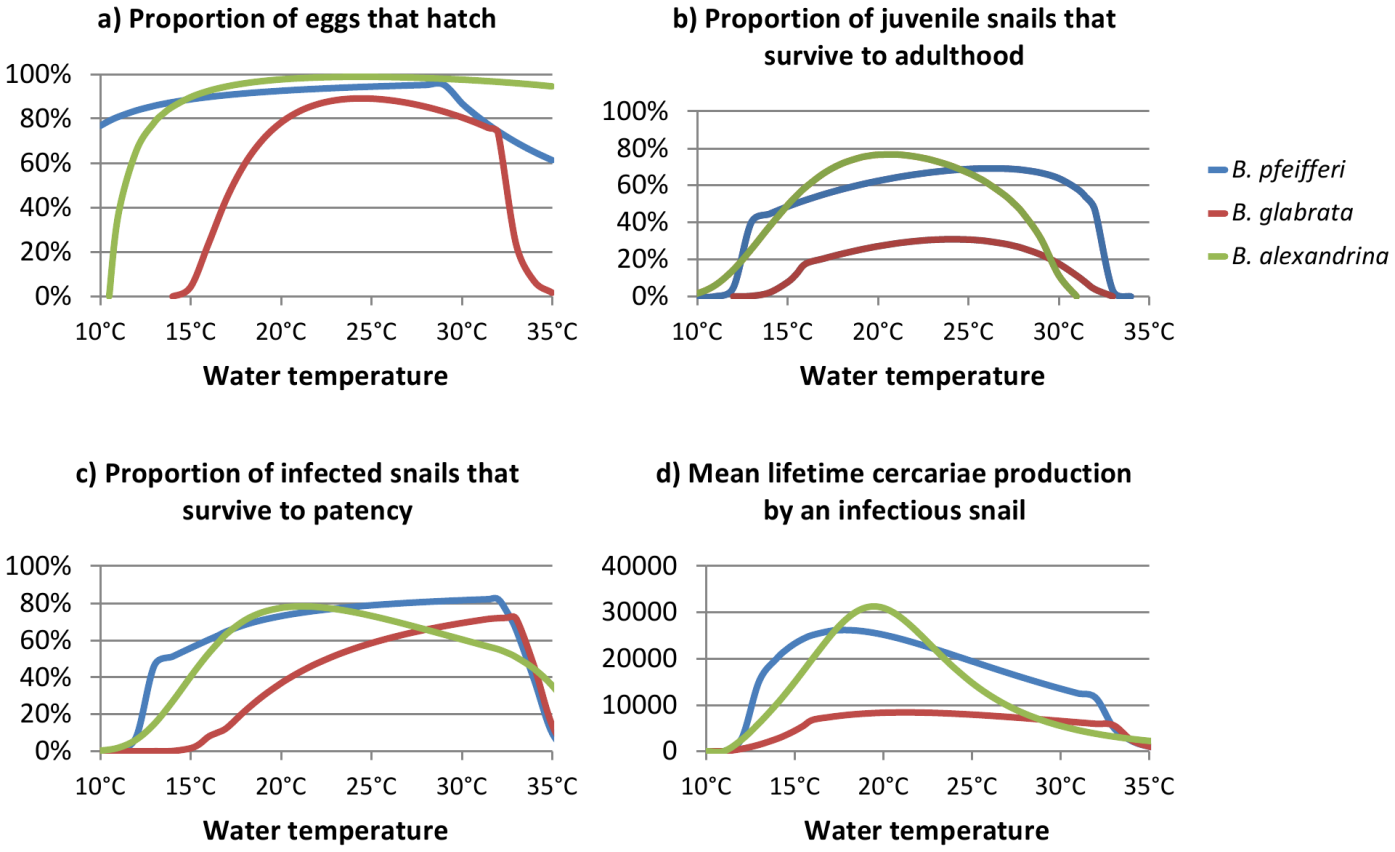
Proportions of eggs and snail surviving each stage, and cercariae production by infected snails. a) Proportion of eggs laid that hatch. b) Proportion of juvenile snails that survive to adulthood (assuming no additional density dependent mortality, and that no juvenile snails develop patent infections before they become adults). c) Proportion of infected snails that survive to patency (assuming no additional density dependent mortality). d) Median lifetime cercariae production by an infectious snail (assuming no additional density dependent mortality). Results are shown for the lower mortality scenarios. Blue lines show *B. pfeifferi*, red lines show *B. glabrata* and green lines show *B. alexandrina.*

The proportion of simulated snail eggs that hatch is high for both *B. pfeifferi* and *B. alexandrina* at all moderate temperatures (greater than 80% between 11.0°C and 31.0°C for *B. pfeifferi* and 13.5°C and >35°C *B. alexandrina*) (figure 4a). The proportion is lower for *B. glabrata* at all temperatures, with less than 60% of eggs hatching at temperatures below 18.0°C and above 30.0°C. Assuming that mortality rates do not increase as a result of high snail densities or patent infections, over 50% of simulated *B. pfeifferi* and *B. alexandrina* juveniles will survive to become adults at temperatures between 15.5°C–31.5°C and 15.5°C-27.5°C respectively (figure 4b). Survival to adulthood is much lower for *B. glabrata*, with a maximum of 31% of snails surviving (at 23.0°C to 25.0°C).

The proportion of infected snails that survive to develop patent infections increases gradually with increasing temperature for both *B. pfeifferi* and *B. glabrata* (figure 4c), however the proportion surviving is higher for *B. pfeifferi* at all temperatures. Assuming no density dependent increase in mortality rates, a maximum of 82% of *B. pfeifferi* survive the prepatent period (at 32.0°C), compared to 60% of *B. glabrata* (at 32.5°C). The proportion of *B. alexandrina* surviving prepatency increases with increasing temperature up to 78% at 21.0°C, before falling again.

Figure 4d shows the median number of cercariae produced by each infectious snail during its lifetime, assuming no density dependent mortality. The median number per infectious snail peaks at 26,300 at 18.0°C for *B. pfeifferi* and at 31,300 at 19.5°C for *B. alexandrina*. It remains above 80% of its maximum between 14.5°C and 23.5°C for *B. pfeifferi* and 17.5°C and 22.0°C for *B. alexandrina*. Median numbers are much lower for *B. glabrata*, and vary less with temperature, peaking at 8,400 at 21.0°C, and remaining above 80% of their maximum between 16.0°C and 29.0°C.

## Discussion

In areas currently at risk for schistosomiasis, temperatures are projected to increase by 2°C to 5°C by 2090–2099, relative to temperatures in 1980–1999[26]. There is some evidence that rising temperatures may already be affecting the distribution of schistosomiasis[2]–[4]. Previous attempts to predict the effects of increasing temperatures on schistosomiasis have largely neglected the issue of multiple species of intermediate host snail[9]–[12]. This is the first study to look at the effects of parameterising a dynamical model of schistosomiasis and temperature to different species of snail.

The results suggest that *B. alexandrina* may be better adapted to slightly cooler water temperatures than *B. pfeifferi*, as both the maximum and minimum temperatures at which the simulated populations could survive indefinitely were lower. Simulated *B. glabrata* populations could survive indefinitely at a smaller range of temperatures than either *B. pfeifferi* or *B. alexandrina* populations, and died out at constant temperatures outside 17.0°C to 29.5°C. Doubling snail mortality rates slightly reduced the range of temperatures at which *B. pfeifferi* and *B. alexandrina* populations could survive, and slightly reduced mean population size at most temperatures. Doubling mortality rates had a greater effect on *B. glabrata*, reducing the range of temperatures at which the population could survive to only 23.0°C to 26.5°C and reducing maximum population size by 29%.

Compared to when *B. pfeifferi* were simulated, infection risk peaked at a slightly higher temperature when *B. alexandrina* were simulated (19.0°C compared to 16.5°C), and at a higher temperature still when *B. glabrata* were simulated (25.0°C). Doubling mortality rates reduced the temperature at which infection risk peaked to 15.0°C when *B. pfeifferi* were simulated, but had no effect on the temperature at which infection risk peaked when *B. glabrata* and *B. alexandrina* were simulated. Absolute infection risk was lower at most temperatures when *B. alexandrina* were simulated, compared to when *B. pfeifferi* were simulated, and much lower at all temperatures when *B. glabrata* were simulated. Doubling snail mortality rates more than halved absolute infection risk for all three species at all temperatures.

Our findings suggest that in most areas where *B. pfeifferi* is the main intermediate host for *S. mansoni,* infection risk will fall as temperatures increase. Infection risk may increase dramatically at the cooler limits of *B. pfeifferi*'s range however. In general, the results suggest that infection risk will also fall in many areas where *B. alexandrina* is the main intermediate host, although it may increase substantially in areas and/or seasons where mean temperatures are currently below around 19.0°C. Where *B. glabrata* is the main intermediate host, infection risk may increase in a much higher proportion of areas and/or seasons, however increases in risk may be smaller. The exception to this is if *B. glabrata* spreads to areas that are currently too cold for it, as infection risk is relatively high even at the coolest temperatures suitable for *B. glabrata*. We will explore this further in future work, running the models using water temperatures from climate projections in regions where the snail species are found. This will also enable us to validate the models by comparing model output for the present day with empirical data from *S. mansoni* prevalence surveys.

The models also suggest that, all else being equal, infection risk may be lower in areas where *B. glabrata* is the intermediate host than in areas where *B. pfeifferi* or *B. alexandrina* are the intermediate host. These results should be interpreted with caution, however, for two reasons. The first is that much of the difference in absolute infection risk is due to the fact that at moderate temperatures simulated *B. glabrata* have much higher mortality rates than simulated *B. pfeifferi* and *B. alexandrina* (figure 2e), resulting in fewer snails surviving the prepatent period (figure 4c), and in lower median lifetime cercariae production by infectious snails (figure 4d). Absolute infection risks in the *B. glabrata* model with standard mortality rates are much closer to absolute infection risks in the *B. pfeifferi* and *B. alexandrina* models with mortality rates doubled. Mortality rates in the model are estimated from mortality rates in laboratory experiments however, and it is therefore possible that the much higher mortality rates of *B. glabrata* were due to different experimental conditions only, and not due to genuine differences between the species.

Secondly, the model does not take into account differences between snail species in susceptibility to *S. mansoni* infection or in cercariae production rates. This is because susceptibility appears to vary as greatly within snail species as it does between species, and there is large variation between individual snails of the same species in cercariae production rates (see Model Parameterisation S1). This should have no effect on the relationship between water temperature and relative infection risk, and very little effect on numbers of snails, but could have had a large effect on absolute infection risk in the models.

The model was parameterised using the best available data for each snail species. We consider there are two main limitations of the data. First, in the vast majority of the experiments that informed model parameterisation, snails were identified using morphological methods only. This means that it is possible that not all of the data were from the species of snail that they were reported to be from, as there can be discrepancies between the molecular and morphological classification of *Biomphalaria* specimens[27]. Second, experimental conditions (e.g. snail densities and feeding strategy) will have varied between the different experiments that produced the data used to parameterise the model. The effect of these differences has been minimised by using data from one experiment only to fit each model parameter wherever possible. This means that the overall relationship between water temperature and snail numbers and relative infection risk should not have been affected greatly by the different experimental conditions. It is important to note that experimental differences may have had a larger effect on absolute snail numbers and infection risk.

The results for all three species suggest that areas currently too cold for substantial schistosome transmission need to be monitored to prevent future epidemics occurring. Uninfected *B. pfeifferi* and *B. alexandrina* may be found in these areas already, whereas *B. glabrata* may need to spread to them from nearby, warmer areas. In areas where schistosomiasis is currently found, the species of intermediate host snail is likely to be important in determining the effects of increasing temperatures on schistosomiasis transmission. Schistosomiasis risk is likely to decrease in the majority of areas where *B. pfeifferi* is the intermediate host. Where *B. glabrata* is the host, risk may increase in some areas, but the increase in risk is unlikely to be large. In some areas where *B. alexandrina* is the intermediate host however, risk may more than double with only a 2°C increase in temperature. These findings highlight the importance of taking a more detailed and nuanced approach to modelling the association between climate change and schistosomiasis. Our approach can also be used as a framework for similar types of investigation into other parasites with life-cycles that include temperature sensitive stages and/or intermediate hosts.

## Supporting Information

Model Parameterisation S1 **Additional information on the data and equations used to parameterise the model to** ***B. glabrata*** **and** ***B. alexandrina*** **snails.**
(DOCX)

## References

[1] McCreesh N, Booth M (2013) Challenges in predicting the effects of climate change on *Schistosoma mansoni* and *Schistosoma haematobium* transmission potential. Trends Parasitol 29: 548–555. 24064438 10.1016/j.pt.2013.08.007

[2] Kabatereine NB, Brooker S, Tukahebwa EM, Kazibwe F, Onapa AW (2004) Epidemiology and geography of *Schistosoma mansoni* in Uganda: implications for planning control. Trop Med Int Health 9: 372–380. 14996367 10.1046/j.1365-3156.2003.01176.x

[3] Rubaihayo J, Moghusu E, Clouds P, Abaasa A (2008) Schistosomiasis transmission at high altitude crater lakes in Western Uganda. BMC Infect Dis 8: 110. 18694485 10.1186/1471-2334-8-110 PMC2518556

[4] Lachish T, Tandlich M, Grossman T, Schwartz E (2013) High rate of schistosomiasis in travelers after a brief exposure to the high altitude Nyinambuga Crater Lake (1630 m), Uganda. Clin Infect Dis. 10.1093/cid/cit559 24021485

[5] Stensgaard A-S, Utzinger J, Vounatsou P, Hürlimann E, Schur N, et al. (in press) Large-scale determinants of intestinal schistosomiasis and intermediate host snail distribution across Africa: Does climate matter? Acta Trop 10.1016/j.actatropica.2011.11.010 22142789

[6] Brown D (1994) Freshwater Snails Of Africa And Their Medical Importance. London, UK: Taylor & Francis.

[7] Appleton CC (1978) Review of literature on abiotic factors influencing the distribution and life cycle of bilharzia intermediate host snails. Malacological Review 11: 1–25.

[8] Zhou XN, Yang GJ, Yang K, Wang XH, Hong QB, et al (2008) Potential impact of climate change on schistosomiasis transmission in China. Am J Trop Med Hyg 78: 188–194. 18256410

[9] Mangal TD, Paterson S, Fenton A (2008) Predicting the impact of long-term temperature changes on the epidemiology and control of schistosomiasis: a mechanistic model. PLoS One 3: e1438. 18197249 10.1371/journal.pone.0001438 PMC2190776

[10] Mangal TD (2009) Developing spatio-temporal models of schistosomiasis transmission with climate change [PhD]: University of Liverpool.

[11] Martens WJM, Jetten TH, Rotmans J, Niessen LW (1995) Climate change and vector-borne diseases: A global modelling perspective. Global Environ Change 5: 195–209.

[12] Martens WJM, Jetten TH, Focks DA (1997) Sensitivity of malaria, schistosomiasis and dengue to global warming. Clim Change 35: 145–156.

[13] McCreesh N, Booth M (2014) The Effect of Increasing Water Temperatures on *Schistosoma mansoni* Transmission and *Biomphalaria pfeifferi* Population Dynamics: An Agent-Based Modelling Study. PLoS One 9(7): e101462. doi:10.1371/journal.pone.0101462 10.1371/journal.pone.0101462 PMC4079709 24987963

[14] Thomas JD, Tait AI (1984) Control of the snail hosts of schistosomiasis by environmental manipulation: A field and laboratory appraisal in the Ibadan area, Nigeria. Philosophical Transactions of the Royal Society of London B, Biological Sciences 305: 201–253. 6144118 10.1098/rstb.1984.0056

[15] Appleton C (1977) The freshwater Mollusca of Tongaland, with a note on molluscan distribution in Lake Sibaya. Annals of the Natal Museum 23: 129–144.

[16] Dupouy J, Mimpfoundi R (1986) Cycle biologique de *Biomphalaria pfeifferi* (Krauss) dans les milieux anthropisés du district de Yaoundé (Cameroun). Compte rendu des séances de la société de biogéographie 62: 47–60.

[17] Wibaux-Charlois M, Yelnik A, Ibrahima H, Same-Ekobo A, Ripert C (1982) Etude épidémiologique de la bilharziose à *Schistosoma haematobium* dans le périmètre rizicole de Yagoua (nord Cameroun). Distribution et écologie des hôtes intermédiaires. Bull Soc Pathol Exot Filiales 75: 72–93. 7201892

[18] Gryseels B (1985) La répartition de *Biomphalaria* et la transmission de *Schistosoma* dans la plaine de la Ruzizi, Burundi: étude préliminaire. Ann Soc belge Méd trop 65: 49–58. 4004374

[19] Madsen H, Daffalla A, Karoum K, Frandsen F (1988) Distribution of freshwater snails in irrigation schemes in the Sudan. J Appl Ecol: 853–866.

[20] Dennis E, Vorkpor P, Holzer B, Hanson A, Saladin B, et al (1983) Studies on the epidemiology of schistosomiasis in Liberia: the prevalence and intensity of schistosomal infections in Bong County and the bionomics of the snail intermediate hosts. Acta Trop 40: 205. 6138973

[21] Doumenge J, Mott K, Cheung C, Villenave D (1987) Atlas of the global distribution of schistosomiasis. Bordeaux, France: Presses Universitaires de Bordeaux.

[22] Dazo BC, Hairston NG, Dawood IK (1966) The ecology of *Bulinus truncatus* and *Biomphalaria alexandrina* and its implications for the control of bilharziasis in the Egypt-49 project area. Bull World Health Organ 35: 339–356. 5297630 PMC2476091

[23] Paraense WL (2001) The schistosome vectors in the Americas. Mem Inst Oswaldo Cruz 96 Suppl: 7–16 10.1590/s0074-02762001000900002 11586421

[24] Sturrock RF (1973) Field studies on the transmission of *Schistosoma mansoni* and on the bionomics of its intermediate host, *Biomphalaria glabrata*, on St. Lucia, West Indies. Int J Parasitol 3: 175–194. 4706570 10.1016/0020-7519(73)90023-4

[25] Jobin WR (1970) Population dynamics of aquatic snails in three farm ponds of Puerto Rico. Am J Trop Med Hyg 19: 1038–1048. 5493050 10.4269/ajtmh.1970.19.1038

[26] IPCC (2007) Summary for Policymakers. Cambridge, United Kingdom and New York, NY, USA.

[27] Plam M, Jørgensen A, Kristensen TK, Madsen H (2008) Sympatric *Biomphalaria* species (Gastropoda: Planorbidae) in Lake Albert, Uganda, show homoplasies in shell morphology. Afr Zool 43: 34–44.

