# Supplementary material for: The Effect of Simulating Different Intermediate Host Snail Species on the Link between Water Temperature and Schistosomiasis Risk
Source: PLoS One. 2014 Jul 2;9(7):e87892. doi: 10.1371/journal.pone.0087892 (PMC4079337; doi:10.1371/journal.pone.0087892)
Supplement: Model Parameterisation S1 — Additional information on the data and equations used to parameterise the model to B. glabrata and B. alexandrina snails. (DOCX) [file pone.0087892.s001.docx]

**Model parameterisation S1**

Snail and parasite birth or production, development, mortality and infection rates in the model are temperature-dependent. Data on these rates were collected from a wide range of empirical studies. To calculate the rates used in the model, linear, quadratic, exponential, Weibull, Lactin[[1](#_ENREF_1)], Gompertz or piecewise equations were fitted to the data points from the empirical studies using a least squares method. The choices of equations to use was based on knowledge of which relationships were biologically plausible, and on looking at plots of the data. Table S1 gives details of the rates that are common to the models of all three snail species (*B. pfeifferi*, *B. glabrata* and *B. alexandrina*). Table S2 gives details of all of the rates used in the *B. glabrata* model, and Table S3 gives details of the rates used in the *B. alexandrina* model.

**Juvenile development**

*B. glabrata*

Data on the number of days between hatching and the start of egg laying at 20°C, 25°C and 30°C were available from one laboratory study[[2](#_ENREF_2)]. They were converted into heat units gained per hour, with 100 heat units needed for the juvenile snails to become adults (Figure S1a). This was done by dividing 100 by the number of hours between hatching and the start of egg laying. A ‘heat unit’ approach allows the degree of development of the snail to be tracked in the model if fluctuating temperatures are simulated, and, unlike a degree-day approach, does not make an assumption of linearity[[3](#_ENREF_3)]. It was assumed that no juvenile development occurs above and below the estimated maximum and minimum temperatures for *B. pfeifferi* juvenile development[[4](#_ENREF_4)]. These were 6.1°C and 33.6°C respectively. A Lactin curve was fitted through the three empirical data points, subject to the constraint that the values the curve took at 6.1°C and 33.6°C were less than or equal to zero, and this curve was used to simulate juvenile *B. glabrata* development in the model. It was assumed that no development occurs above and below 33.6°C and 6.5°C respectively, the temperatures at which the curve met the x-axis.

*B. alexandrina*

Data on the number of days between hatching and the start of egg laying were available from one laboratory study at 20°C, 25°C, 28°C and 30°C[[5](#_ENREF_5)], and from a second laboratory study at 18°C, 26°C and 28°C[[6](#_ENREF_6)]. They were converted into heat units gained per hour and a Lactin equation fitted through the seven points was used to simulate juvenile *B. alexandrina* development in the model (Figure S1b). It was assumed that no development occurs above and below 31.1°C and 5.2°C respectively, the temperatures at which the curve met the x-axis.

*B. glabrata* and *B. alexandrina*

Field[[7](#_ENREF_7)] and laboratory[[8](#_ENREF_8)] studies in *B. pfeifferi* found that high temperatures during a juvenile snail’s development period (greater than 17-39 degree hours above 27°C a day) could result in permanent reduced egg production in adults. To our knowledge, no similar studies have been conducted with *B. glabrata* or *B. alexandrina*. The same relationship between high temperatures in development and reduced adult egg production was therefore simulated for *B. glabrata* and for *B. alexandrina* as for *B. pfeifferi*.

In the model, a record is kept for each juvenile snail of the number degree hours spent above 27°C, above a threshold of 39 degree hours above 27°C a day. For instance, if a model day contained 48 degree hours above 27°C then the snail’s total number of degree hours above 27°C would be increased by 48 - 39 = 9 degree hours. On days where there are ≤39 degree hours above 27°C, the total number of degree hours above 27°C does not increase. When the snail becomes an adult, its high temperature egg production weight is calculated. If the number of degree hours above 27°C was ≤39 degree hours every day during its development, the weight takes the value one. If the number of degree hours above 27°C was >39 degree hours on any day, then the value of the weight is determined from an exponential line fitted to the data from the laboratory study of *B. pfeifferi*[[8](#_ENREF_8)] (Figure S1c).


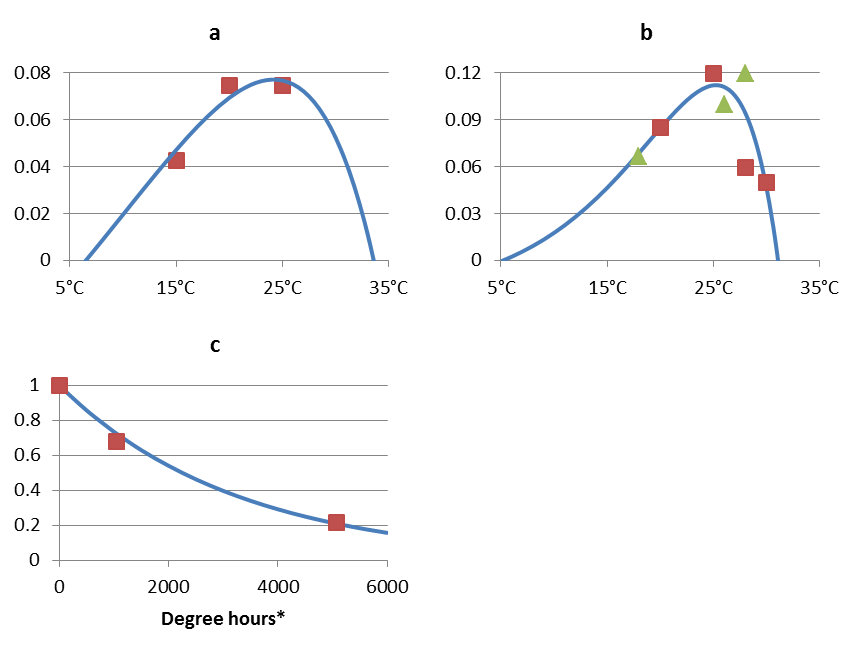


**Figure S1. Juvenile development rate graphs.** a) and b) Rate of juvenile heat unit gain per hour by water temperature. 100 heat units are necessary to complete development and start egg laying. a) *B. glabrata*. Red squares show data from a laboratory study[[2](#_ENREF_2)]. The blue line shows a Lactin model fitted through the data, and is used to determine juvenile development rates in the model. b) *B. alexandrina*. Red squares[[5](#_ENREF_5)] and green triangles[[6](#_ENREF_6)] show data from laboratory studies. The blue line shows a Lactin model fitted through the data, and is used to determine juvenile development rates in the model. c) Effect of high temperatures during juvenile development on the adult egg production rate. Red squares show data from a laboratory study of *B. pfeifferi*[[8](#_ENREF_8)] and the blue lines shows the relationship used in the model for all three snail species. *Cumulative number of degree hours above 27°C, above a threshold of 39 degree hours above 27°C per day

**Egg production**

*B. glabrata*

A laboratory study recorded the mean number of eggs produced per snail per day by adult *B. glabrata* kept at 17.5°C, 20.0°C, 22.5°C, 25.0°C and 27.5°C[[9](#_ENREF_9)]. This was converted into eggs per snail per hour and a Lactin equation was fitted through the five data points (Figure S2a). This equation gave an estimated egg production rate at 23°C of 19 eggs per snail per day. This is much higher than the estimated 6 eggs per snail per day laid by snails in a pond with an average temperature of 23°C[[10](#_ENREF_10)], and therefore the equation was scaled by 6/19 at all temperatures. No egg production occurred in the model above 30.4°C and below 11.7°C, the temperatures at which the Lactin equation met the x-axis.

As for *B. pfeifferi*, egg production in the model was reduced by a further 90% to simulate temperature independent egg mortality before and during hatching.

Experimental data suggest that there is little or no difference in egg production rates between uninfected and prepatent *B. glabrata* kept at 25°C at seven or 14 days post-exposure[[11](#_ENREF_11)]. Egg production in prepatent snails compared to uninfected snails dropped sharply from 21 days post-exposure however. Patency was reached at around 28 days. In another study, prepatent snails ceased producing eggs entirely five weeks post-exposure[[12](#_ENREF_12)]. In the model, prepatent snails stop producing eggs after they had accumulated 50% of the heat units necessary to become infectious.

*B. alexandrina*

Experimental data were available on the number of eggs produced per snail per day by adult *B. alexandrina* kept at six temperatures between 12.5°C and 30°C[[5](#_ENREF_5)]. The same study found that no egg production occurred at 10°C or 35°C. A Lactin equation fitted through the six points was used to determine egg production rates in the model (Figure S2b). No egg production occurred in the model above 30.1°C and below 10.0°C, the temperatures at which the Lactin equation met the x-axis.

For both *B. pfeifferi* and *B. glabrata*, simulated egg production rates were set 90% lower than estimated egg production rates to incorporate temperature independent egg mortality before and during hatching. The egg production rates estimated for *B. alexandrina* from the experimental data described above were 8-22 times lower than the *B. alexandrina* egg production rates found in another study however[[13](#_ENREF_13)]. The maximum rate was also 8.6 and 9.5 times lower than the maximum rates estimated for *B. pfeifferi* and *B. glabrata* respectively. Furthermore, the simulated *B. alexandrina* population very quickly died out at all temperatures if simulated egg production rates are reduced by 90%. For these reasons, egg production rates were set equal to the rates estimated using the Lactin equation described above, and were not reduced.

No data were available on egg production by prepatent or patently infected *B. alexandrina*. Both *B. pfeifferi* and *B. glabrata* cease to produce eggs roughly halfway through their prepatent periods however, and therefore simulated prepatent *B. alexandrina* stop producing eggs after they had accumulated 50% of the heat units necessary to become infectious.

**Figure S2. Egg production graphs.** Eggs/snail/hour. a) *B. glabrata.* The green triangles show empirical data from a laboratory studies[[9](#_ENREF_9)]. The purple cross shows empirical data from a field study[[10](#_ENREF_10)]. The blue line is a Lactin model fitted to the laboratory data. The red line is the Lactin model scaled to pass through the field data point. b) *B. alexandrina*. Red squares show data from a laboratory study[[5](#_ENREF_5)]. The blue line shows a Lactin model fitted through the data, and is used to determine egg production rates in the model.

**Egg development**

*B. glabrata*

A laboratory study recorded the number of days until hatching, and the proportion of eggs that hatched, for eggs from pigmented and unpigmented *B. glabrata* kept at 11 temperatures between 14°C and 34°C[[14](#_ENREF_14)]. There was very little difference between pigmented and unpigmented snails in either development or mortality rates, and the data from the pigmented snails was therefore used.

Days until hatching were converted into heat unit gain per hour (with 100 heat units needed for hatching) and a Lactin equation was fitted through the points (Figure S3a). This equation was used to simulate egg development rates in the model. No egg development was assumed to occur below 13.8°C and above 40.0°C in the model, where the fitted line meets the x-axis.

Data on the proportion of eggs that hatched and the number of days to hatching were used to calculate the mortality rate per hour at each temperature. Mortality rates were less than 0.0021 per hour at all temperatures except 34°C. A quadratic equation was fitted through these points and used to calculate egg mortality rates in the model at all temperature ≤32°C (Figure S3c). The mortality rate was much higher at 34°C (0.017 per hour). A linear equation was fitted between the empirical morality rates at 32°C and 34°C and this was used to calculate mortality rates in the model at all temperatures >32°C.

*B. alexandrina*

Data were available from a laboratory study on the number of days until hatching and the proportion of eggs that hatched for *B. alexandrina* eggs kept at 12.5°C, 15°C, 20°C, 25°C, 30°C and 35°C[[5](#_ENREF_5)]. No eggs hatched at 12.5°C or 35°C. The number of days until hatching was converted into heat unit gain per hour and a quadratic equation fitted through the points (Figure S3b). This was used to determine egg heat unit gain in the model. No egg development occurs in the model below 10.1°C where the line meets the x-axis. Data on the proportion of eggs that hatched and the number of days to hatching were used to calculate the mortality rate per hour at each temperature between 15°C and 30°C (Figure S3d). A quadratic equation was fitted through these points and used to simulate egg mortality rates in the model.

**Figure S3. Egg development and mortality graphs.** a) *B. glabrata* egg heat unit gain per hour. 100 heat units are needed for hatching. Red squares show data from a laboratory study[[14](#_ENREF_14)]. The blue line shows a Lactin model fitted through the data, and is used to determine egg development rates in the model. b) *B. alexandrina* egg heat unit gain per hour. 100 heat units are needed for hatching. Red squares show data from a laboratory study[[5](#_ENREF_5)]. The blue line shows a quadratic equation fitted through the data, and is used to determine egg development rates in the model. c) *B. glabrata* egg mortality rates per hour. Red squares show data from a laboratory study[[14](#_ENREF_14)]. The blue line shows a piecewise quadratic and linear equation fitted through the data, and is used to determine egg mortality rates in the model. d) *B. alexandrina* egg mortality rates per hour. Red squares show data from a laboratory study[[5](#_ENREF_5)]. The blue line shows a quadratic equation fitted through the data, and is used to determine egg mortality rates in the model.

**Mortality**

*B. glabrata*

No suitable data were available on the mortality rates of uninfected *B. glabrata* at different water temperatures. Experimental data were available on the mortality rates of *B. glabrata* with prepatent *S. mansoni* infections at 14 temperatures between 16°C and 36°C[[15](#_ENREF_15)] however, and studies suggest that there is little or no difference between mortality rates in uninfected and prepatent *B. glabrata*[[12](#_ENREF_12),[16](#_ENREF_16)]. These mortality rates were therefore used to estimate mortality rates for both prepatent and uninfected snails.

The data suggested that water temperature has little or no effect on mortality rates at moderate temperatures of between 16°C-33°C[[15](#_ENREF_15)]. The mean mortality rate between these temperatures was therefore calculated and used to simulate moderate temperature mortality rates in the model (Figure S4a). Laboratory data were available on *B. glabrata* mortality rates at higher temperatures: at 34°C[[15](#_ENREF_15)], 35°C[[15](#_ENREF_15)], and 40°C[[14](#_ENREF_14)]. An exponential line was fitted through these three points and the moderate temperature mortality rate at 33°C (Figure S4b).

For lower temperatures, data were available at 0°C only[[14](#_ENREF_14)]. An exponential line was fitted through this point and the moderate temperature mortality rate at 16°C.

*B. alexandrina*

El-Hassan recorded the proportions dead after two weeks for uninfected juvenile and adult *B. alexandrina* kept at seven temperatures between 10°C and 37°C[[5](#_ENREF_5)]. There was very little difference between the mortality of juvenile and adult snails, and therefore the mean proportion dead at each temperature was calculated. 100% of snails died within two weeks at 37°C. Mortality rates were calculated for each other temperature, and a quadratic equation fitted to them (Figure S4c). This was used to calculate the mortality rates for uninfected snails at all temperatures in the model.

No suitable data were available on mortality rates in prepatent and infected *B. alexandrina*. Prepatent *B. glabrata* show no increase in mortality compared to uninfected controls however[[16](#_ENREF_16)], and therefore no increase in mortality in prepatent *B. alexandrina* was simulated.

*B. glabrata* and *B. alexandrina*

No suitable data were available on the mortality rates of infectious *B. glabrata* or *B. alexandrina* compared to the mortality rates of uninfected *B. glabrata* or *B. alexandrina* at different water temperatures. Data were available, however, on mortality rates in shedding and non-shedding *B. pfeifferi* at four temperatures between 23-28°C[[17](#_ENREF_17)] (Figure S4d). Mortality rates were 2.1-6.6 times higher in shedding snails, with the ratio increasing with increasing temperature. It was assumed that the ratio was equal to one below 11.2°C, the estimated temperature below which *B. pfeifferi* do not produce cercariae[[4](#_ENREF_4)]. An exponential line was fitted between the four data points and the estimated point at 11.2°C, and this was used to model increased mortality in infectious *B. glabrata* and *B. alexandrina* compared to uninfected *B. glabrata* and *B. alexandrina* at temperatures above 11.2°C. Below 11.2°C the modelled ratio was equal to one.

In the *B. pfeifferi* model[[4](#_ENREF_4)], mortality rates from laboratory data were multiplied by 1.35 (estimated from field data[[18](#_ENREF_18)]) to account for increased mortality rates in a natural setting. Data from field studies of *B. glabrata* populations suggest that *B. glabrata* mortality rates may be between 2.08[[10](#_ENREF_10)] and 2.17[[19](#_ENREF_19)] times higher in a natural setting than in a laboratory. Data from a field study of *B. alexandrina* suggest that *B. alexandrina* field mortality rates may be 10.05 times greater than laboratory mortality rates[[20](#_ENREF_20)]. Field mortality rates are estimated using indirect methods however, and will vary greatly between different locations and seasons due to differences in factors such as predation, inter-specific competition, and water quality. To improve comparisons between model outputs for the different snail species, it is therefore assumed in the models for all three species that field mortality rates are 1.35 times higher than rates estimated from laboratory data.

**Figure S4. Snail mortality rates.** Mortality rates per hour. a) *B. glabrata* 16°C to 33°C. The red squares show data from a laboratory study[[15](#_ENREF_15)] and the blue line shows the mean mortality rate between 16°C and 33°C. b) *B. glabrata* all temperatures. The red squares[[15](#_ENREF_15)] and green triangles[[14](#_ENREF_14)] show data from laboratory studies and the blue line shows an equation fitted through the points. A scaled version of the equation shown by the blue line was used to determine uninfected and prepatent snail mortality rates in the model. c) *B alexandrina*. The red squares show data from a laboratory study[[5](#_ENREF_5)] and the blue line shows a quadratic line fitted through the points. A scaled version of the equation shown by the blue line was used to determine uninfected and prepatent snail mortality rates in the model. d) Mortality rates in infectious snails relative to mortality rates in uninfected snails. The blue line shows the relationship used in the model for all three snail species. The red squares show experimental data from *B. pfeifferi*[[17](#_ENREF_17)]. The green triangle shows the estimated temperature below which *B. pfeifferi* do not produce cercariae.

**Snail density dependence**

Experimental data suggest that high snail densities have a greater effect on egg production rates than on mortality rates in both *B. glabrata*[[21](#_ENREF_21)] and *B. alexandrina*[[22](#_ENREF_22)]. This is also the case for *B. pfeifferi*[[4](#_ENREF_4)]. The same relationships between snail numbers and egg production and mortality rates were therefore used for all three snail species. In the model, it is assumed that the environment can support up to 300 snails with no negative effect on fecundity. Above this number, the rate of egg production drops following a Gompertz distribution (figure S5a). At population numbers of more than 600 snails, juvenile and adult mortality rates also increase (figure S5b).


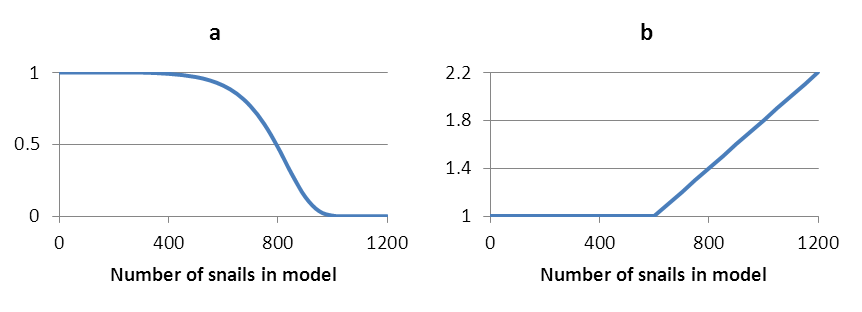


**Figure S5. Effect of large numbers of simulated snails on simulated egg production and mortality rates.** a) Simulated drop in egg production rates at high snail numbers. b) Simulated increase in snail mortality rates at high snail numbers.

**Parasite development within the snail**

*B. glabrata*

Pflüger measured the time to patency in *S. mansoni* infected *B. glabrata* kept at 14 constant temperatures between 16°C and 36°C[[15](#_ENREF_15)]. These times were converted into heat units gained per hour. Between 16°C and 32°C there is a positive linear relationship between water temperature and heat unit gain and a linear equation fitted through the points was used to determine parasite development rates in *B. glabrata* in the model. Above 32°C, development rates start to decrease with increasing temperature. This was simulated in the model using a quadratic equation fitted to the data points (figure S6).

Pflüger also measured prepatency times at different fluctuating temperature regimes for *S. mansoni* in *B. glabrata*[[23](#_ENREF_23)]. He found that the assumption of a linear relationship between temperature and development rate does not hold for temperature regimes incorporating temperatures below approximately 16°C, with development periods being shorter than would be expected at low temperatures. A non-linear relationship was therefore modelled for temperatures less than 17°C.

*B. alexandrina*

Very little data were available on the length of prepatency of *S. mansoni* in *B. alexandrina* at different water temperatures. One study recorded a prepatency period in susceptible snails kept at 25°C of 27 days[[24](#_ENREF_24)]. Another recorded a prepatency period of 22-28 days at 26-28°C, depending on the strain of *S. mansoni* used[[25](#_ENREF_25)]. These studies suggest that the duration of prepatency in *B. alexandrina* is closer to the duration of prepatency in *B. pfeifferi* than the duration of prepatency in *B. glabrata*. The same relationship between temperature and parasite heat unit gain was therefore used for *B. alexandrina* as for *B. pfeifferi*[[4](#_ENREF_4)].

**Figure S6. Parasite development within *B. glabrata*.** Rate of heat unit gain per hour, with 100 heat units needed for the snail to become infectious. The blue line shows the relationship used in the model. The red squares show laboratory data[[15](#_ENREF_15)]. Laboratory data for temperatures between 16°C and 32°C are not shown as they were given in the form of a fitted equation in the original paper[[15](#_ENREF_15)]

**Miracidia and cercariae aging and mortality**

As miracidia and cercariae are free living stages the species of snail involved in maintaining the lifecycle is unlikely to have any effect on their mortality rates or decrease in infectiousness with age. The same rates were therefore used for *B. glabrata* and *B. alexandrina* as were used for *B. pfeifferi*[[4](#_ENREF_4)].

**Susceptibility to infection and cercariae production**

The rate of infection of snails by miracidia in the model is a function of the miracidiums’ biological ages, water temperature and the number of snails in the model[[4](#_ENREF_4)]. It does not depend on the species of snail. This is because the susceptibility of *Biomphalaria* snails to *S. mansoni* infection varies as greatly within species as it does between snail species, and depends on the source of the snails and parasite used in the experiment[[25-28](#_ENREF_25)].

Data on the relationship between water temperature and cercariae production were only available from *B. glabrata*[[29](#_ENREF_29)], and therefore these data were used to simulate cercariae production at different temperatures for *B. glabrata*, *B. alexandrina* and *B. pfeifferi*. Similarly, suitable data on the relationship between time of day and cercariae release were only available from experiments with *B. stanleyi*[[30](#_ENREF_30)], and therefore these data were used to simulate variation in cercariae production by time in all three snail species. Data on absolute numbers of cercariae produced per day by infectious snails show a large amount of variation within the same snail species[[31-33](#_ENREF_31)], and for that reason differences between snail species in the absolute numbers of cercariae produced per day were not incorporated into the model.

| **Rate (per hour) [varies with]** | **Source(s)** | **Range of data** | **Fitted model** | **Equation/ Parameters** | **Behaviour outside range of data** |
| --- | --- | --- | --- | --- | --- |
| Relative increase in mortality in infectious snails [water temperature (*T_w_*)] | Foster[[17](#_ENREF_17)] | 23-28°C | Exponential | 0.3073 * e ^ (0.1002 * *T_w_*) | Line extended backwards to 11.2°C where it takes the value of 1. Below 11.2°C, the relative increase in mortality is taken to be equal to 1. Line extended forwards above 28°C. |
| Relative increase in mortality [number of snails in model *N_s_*] | - | - | Linear | If *N_s_* < 600: 1  If *N_s_* ≥ 600:  (*N_s_* – 600) / 500 + 1 | Lines used for any number of snails in model |
| Relative egg production rate [number of snails in model *N_s_*] | Loreau and Baluku[[34](#_ENREF_34)] | Data from tanks with 8.6 snails/l and 85.7 snails/l only | Gompertz | If *N_s_* < 300: 1  If *N_s_* ≥ 300:  e ^ (0.005 * (1 – e ^ ((*N_s_* -300)/100))) | Lines used for any number of snails in model |
| Relative egg production rate [Cumulative number of degree hours above 27°C, above a threshold of 39 degree hours above 27°C per day, during a juvenile snail’s development period *(d)*] | Appleton[[8](#_ENREF_8)] | 0-5065 degree hours above 27°C, above a threshold of 39 degree hours above 27°C per day | Exponential | e ^ (-0.00031 * *d*) | Line extended to higher values of *d* |
| Miracidium biological age gain [water temperature (*T_w_*)] | Anderson *et al*[[35](#_ENREF_35)] | 5-40°C | Piecewise linear | If *T_w_* < 15°C: 0  If *T_w_* ≥ 15°C:  0.0530 * *T_w_* - 0.349 | No biological age gain occurs below 15°C. The line is extended forwards above 40°C |
| Miracidium mortality rate if *T_w_* ≥ 15°C [miracidium biological age (hours) (*M_a_*)] | Anderson *et al*[[35](#_ENREF_35)] | Miracidium birth to 100% mortality of cohort | Exponential | 1 - (1 - (0.0083 * e ^ ((-0.35 + 0.032 * 25) * *M_a_*))) | Line used to determine mortality rate at all biological ages |
| Miracidium mortality rate if *T_w_* < 15°C [water temperature (*T_w_*)] | Anderson *et al*[[35](#_ENREF_35)] | Miracidium birth to 100% mortality of cohort | Linear | -0.0192 * *T_w_* + 0.2911 | Line used to determine mortality rate for all water temperatures < 15°C |
| Relative miracidium infection rate [miracidium biological age (hours) (*M_a_*)] | Anderson *et al*[[35](#_ENREF_35)] | Miracidium birth to 100% mortality of cohort | Weibull | (0.196 * e ^ ((-2.18 * 10^-6^ / D$6) * *M_a_* ^ 8.33)) / 0.196 | Line used to determine relative infection rate at all biological ages |
| Relative miracidium infection rate [water temperature (*T_w_*)] | Anderson *et al*[[35](#_ENREF_35)] | 15-35°C | Piecewise exponential | If *T_w_* < 15°C: 0  If 15°C ≤ *T_w_* ≤ 25°C:  0.135 * e ^ (0.0572 * *T_w_*)  If *T_w_* > 25°C:  2.28 * e ^ (-0.0544 * *T_w_*) | Lines extended to higher and lower temperatures |
| Relative miracidium infection rate [number of snails in model *N_s_* | - | - | 1 - exponential | 1 – e ^ (-*N_s_* / 3000) | Line used for any number of snails in model |
| Relative cercaria production rate [time of day (hours since midnight *h*)] | Kazibwe[[30](#_ENREF_30)] | 9.00-17.00 | Quadratic | -0.0578 * *h*^2^ + 1.501 * *h* - 8.84 | No cercariae production occurs between 17.00 and 9.00 |
| Cercaria production rate [water temperature (*T_w_*)] | Fried *et al*[[29](#_ENREF_29)] | 12-35°C | Linear | 0.02 * (14.2 * *T_w_* - 159.2) | Line extended in both directions. No cercariae production occurs below 11.2°C where the line meets the x-axis |
| Cercaria biological age gain (hours) [water temperature (*T_w_*)] | Lawson and Wilson[[36](#_ENREF_36)] | 15-40°C | Exponential | 0.199 * e ^ (0.069 * *T_w_*) | Lines extended to higher and lower temperatures |
| Cercaria mortality rate [cercaria biological age (hours) (*C_a_*)] | Lawson and Wilson[[36](#_ENREF_36)] | Cercaria birth to 100% mortality of cohort | Gompertz | 1 – (1 – e ^ (-e ^ (1.75 – 0.056 * *C_a_*))) ^ (*cercaria age gain*) | Line used to determine mortality rate at all biological ages |
| Relative cercaria infection rate [cercaria biological age (hours) (*C_a_*)] | Ghandour *et al*[[37](#_ENREF_37)] | 2-24 hours | Exponential | 0.829 * e ^ (-0.0675 * *C_a_*) | Line used to determine relative infection rate at all biological ages |

**Table S1. Model rates common to all three snail species (*B. pfeifferi*, *B. glabrata* and *B. alexandrina*)**

| **Rate (per hour) [varies with]** | **Source(s)** | **Range of data** | **Fitted model** | **Equation/ Parameters** | **Behaviour outside range of data** |
| --- | --- | --- | --- | --- | --- |
| Juvenile heat unit gain* [water temperature (*T_w_*)] | Sturrock and Sturrock[[2](#_ENREF_2)] | 20-30°C | Lactin | Lactin model with parameters:  ρ = 0.070  λ = -0.14  Δ = 13.8  T_max_ = 38.2°C | Line extended to 6.5°C and 33.6°C where the model meets the x-axis. No heat unit gain occurs outside this range. |
| Egg production rate [water temperature (*T_w_*)] | Pimentel-Souza *et al*[[9](#_ENREF_9)] and Jobin[[10](#_ENREF_10)] | 17.5-27.5°C (laboratory) and 23°C (field) | Lactin | 0.1 * 0.31 * Lactin model with parameters:  ρ = 0.18  λ = -0.35  Δ = 5.4  T_max_ = 31.0°C | Line extended to 11.7°C and 30.4°C where the model meets the x-axis. No egg production occurs outside this range. |
| Egg heat unit gain*  [water temperature (*T_w_*)] | Joubert and Pretorius[[14](#_ENREF_14)] | 14-34°C | Lactin | Lactin model with parameters:  ρ = 0.051  λ = -1.42  Δ = 11.3  T_max_ = 46.0°C | Line extended to 13.8°C and 40.0°C where the model meets the x-axis. No egg heat unit gain occurs outside this range. |
| Egg mortality rate  [water temperature (*T_w_*)] | Joubert and Pretorius[[14](#_ENREF_14)] | 14-34°C | Piecewise quadratic and linear | If *T_w_* ≤ 32°C: 0.000019 * *T_w_*^2^ - 0.00088 * *T_w_* + 0.011  If *T_w_* > 32°C: 0.0074 * *T_w_* - 0.23 | Lines extended in both directions. |
| Uninfected and prepatent snail mortality rate between 16-33°C  [water temperature (*T_w_*)] | Pflüger[[15](#_ENREF_15)] | 16-33°C | Constant | 0.00067 | See rows below |
| Uninfected and prepatent snail mortality rate below 16°C  [water temperature (*T_w_*)] | Joubert and Pretorius[[14](#_ENREF_14)] | 0°C | Exponential | 0.070 * e ^ (-0.29 * *T_w_*) | Line extended to lower temperatures |
| Uninfected and prepatent snail mortality rate above 33°C  [water temperature (*T_w_*)] | Pflüger[[15](#_ENREF_15)] and Joubert and Pretorius[[14](#_ENREF_14)] | 34-40°C | Exponential | 0.000000000000003 * e ^ (0.79 * *T_w_*) | Line extended to higher temperatures |
| Parasite heat unit gain within snail* [water temperature (*T_w_*)] | Pflüger[[15](#_ENREF_15)] and Pflüger[[23](#_ENREF_23)] | 16-36°C | Piecewise linear, exponential and quadratic | If *T_w_* < 17°C:  0.0037 * e ^ ((*T_w_* - 4.7) / 5)  If *T_w_* ≥ 17°C and *T_w_* < 32°C: (100 / 24) * (*T_w_* - 14.2) / 268)  If *T_w_* ≥ 32°C: -0.0080 * *T_w_*^2^ + 0.52 * *T_w_* - 8.18 | Line extended in both directions. No parasite heat unit gain occurs above 38.4°C where the line meets the x-axis |

**Table S2. *B. glabrata* model rates** *Number of heat units necessary to complete stage set to 100

| **Rate (per hour) [varies with]** | **Source(s)** | **Range of data** | **Fitted model** | **Equation/ Parameters** | **Behaviour outside range of data** |
| --- | --- | --- | --- | --- | --- |
| Juvenile heat unit gain* [water temperature (*T_w_*)] | El-Hassan[[5](#_ENREF_5)] and El-Emam[[6](#_ENREF_6)] | 18-30°C | Lactin | Lactin model with parameters:  ρ = 0.159  λ = -0.023  Δ = 6.27  T_max_ = 31.5°C | Line extended to 5.2°C and 31.1°C where the model meets the x-axis. No heat unit gain occurs outside this range. |
| Egg production rate [water temperature (*T_w_*)] | El-Hassan[[5](#_ENREF_5)] | 12.5-30.0°C | Lactin | Lactin model with parameters:  ρ = 0.149  λ = -0.017  Δ = 6.72  T_max_ = 31.1°C | Line extended to 10.0°C and 31.0°C where the model meets the x-axis. No egg production occurs outside this range. |
| Egg heat unit gain*  [water temperature (*T_w_*)] | El-Hassan[[5](#_ENREF_5)] | 12.5-35.0°C | Quadratic | -0.000825 * *T_w_* ^2^ + 0.0576 * *T_w_* - 0.497 | Line extended to 10.1°C, where the model meets the x-axis, and to higher temperatures. No egg heat unit gain occurs below 10.1°C |
| Egg mortality rate  [water temperature (*T_w_*)] | El-Hassan[[5](#_ENREF_5)] | 15.0-35.0°C | Quadratic | 0.0000019 * *T_w_* ^2^ - 0.000091 * *T_w_* + 0.0011 | Line extended to higher and lower temperatures |
| Uninfected and prepatent snail mortality rate  [water temperature (*T_w_*)] | El-Hassan[[5](#_ENREF_5)] | 10.0-37.0°C | Quadratic | 0.0000053 * *T_w_* ^2^ - 0.00020 * *T_w_*  + 0.0021 | Line extended to higher and lower temperatures |
| Parasite heat unit gain within snail* [water temperature (*T_w_*)] | Foster[[17](#_ENREF_17)] and Pflüger[[23](#_ENREF_23)] | 18-32°C (constant temperature) 12-39°C (as part of a fluctuating regime) | Piecewise linear, quadratic and exponential | If *T_w_* < 17°C:  (0.005 * e ^ ((*T_w_* - 4.7) / 5))  If *T_w_* ≥ 17°C and *T_w_* < 32°C:  0.0119 * *T_w_* - 0.143  If *T_w_* ≥ 32°C: 0.87 * (-0.0080 * *T_w_^2^* + 0.52 * *T_w_* - 8.18) | Line extended in both directions. No parasite heat unit gain occurs above 38.4°C where the line meets the x-axis |

**Table S3. *B. alexandrina* model rates** *Number of heat units necessary to complete stage set to 100

**Supporting references**

1. Lactin DJ, Holliday NJ, Johnson DL, Craigen R (1995) Improved Rate Model of Temperature-Dependent Development by Arthropods. Environ Entomol 24: 68-75.

2. Sturrock RF, Sturrock BM (1972) The influence of temperature on the biology of *Biomphalaria glabrata* (Say), intermediate host of *Schistosoma mansoni* on St. Lucia, West Indies. Ann Trop Med Parasitol 66: 385-390.

3. McCreesh N, Booth M (2013) Challenges in predicting the effects of climate change on *Schistosoma mansoni* and *Schistosoma haematobium* transmission potential. Trends Parasitol 29: 548-555.

4. McCreesh N, Booth M The effect of increasing water temperatures on *Schistosoma mansoni* transmission and *Biomphalaria pfeifferi* population dynamics: an agent-based modelling study.

5. El-Hassan AA (1974) Laboratory studies on the direct effect of temperature on *Bulinus truncatus* and *Biomphalaria alexandrina*, the snail intermediate hosts of schistosomes in Egypt. Folia Parasitol (Praha) 21: 181-187.

6. El-Emam MA, Madsen H (1982) The effect of temperature, darkness, starvation and various food types on growth, survival and reproduction of *Helisoma duryi*, *Biomphalaria alexandrina* and *Bulinus truncatus* (Gastropoda: Planorbidae). Hydrobiologia 88: 265-275.

7. Appleton CC (1977) The influence of temperature on the life-cycle and distribution of *Biomphalaria pfeifferi* (Krauss, 1948) in South-Eastern Africa. Int J Parasitol 7: 335-345.

8. Appleton CC, Eriksson IM (1984) The influence of fluctuating above-optimal temperature regimes on the fecundity of *Biomphalaria pfeifferi* (Mollusca: Planorbidae). Trans R Soc Trop Med Hyg 78: 49-54.

9. Pimentel-Souza F, Barbosa ND, Resende DF (1990) Effect of temperature on the reproduction of the snail *Biomphalaria glabrata*. Braz J Med Biol Res 23: 441-449.

10. Jobin WR (1970) Population dynamics of aquatic snails in three farm ponds of Puerto Rico. Am J Trop Med Hyg 19: 1038-1048.

11. Crews AE, Yoshino TP (1989) *Schistosoma mansoni*: Effect of infection on reproduction and gonadal growth in *Biomphalaria glabrata*. Exp Parasitol 68: 326-334.

12. Pan C-T (1963) Generalized and focal tissue responses in the snail, *Australorbis glabratus*, infected with *Schistosoma mansoni*. Ann N Y Acad Sci 113: 475-485.

13. Mangal TD (2009) Developing spatio-temporal models of schistosomiasis transmission with climate change [PhD]: University of Liverpool.

14. Joubert PH, Pretorius SJ (1985) The effect of constant temperatures on the hatching of eggs and survival of the freshwater snail *Biomphalaria glabrata* (say). Journal of the Limnological Society of Southern Africa 11: 75-77.

15. Pfluger W (1980) Experimental epidemiology of schistosomiasis. I. The prepatent period and cercarial production of *Schistosoma mansoni* in *Biomphalaria* snails at various constant temperatures. Z Parasitenkd 63: 159-169.

16. Minchella DJ, Loverde PT (1981) A cost of increased early reproductive effort in the snail *Biomphalaria glabrata*. The American Naturalist 118: 876-881.

17. Foster R (1964) The Effect of Temperature on the Development of *Schistosoma Mansoni* Sambon 1907 in the Intermediate Host. J Trop Med Hyg 67: 289-292.

18. Loreau M, Baluku B (1987) Popoulation dynamics of the freshwater snail *Biomphalaria pfeifferi* in Eastern Zaire. J Molluscan Stud 53: 249-265.

19. Sturrock RF (1973) Field studies on the transmission of *Schistosoma mansoni* and on the bionomics of its intermediate host, *Biomphalaria glabrata*, on St. Lucia, West Indies. Int J Parasitol 3: 175-194.

20. Dazo BC, Hairston NG, Dawood IK (1966) The ecology of *Bulinus truncatus* and *Biomphalaria alexandrina* and its implications for the control of bilharziasis in the Egypt-49 project area. Bull World Health Organ 35: 339-356.

21. Thomas JD, Benjamin M (1974) The effects of population density on growth and reproduction of *Biomphalaria glabrata* (Say) (Gasteropoda: Pulmonata). J Anim Ecol 43: 31-50.

22. Mangal TD, Paterson S, Fenton A (2010) Effects of snail density on growth, reproduction and survival of *Biomphalaria alexandrina* exposed to *Schistosoma mansoni*. Journal of Parasitology Research 2010.

23. Pfluger W (1981) Experimental epidemiology of schistosomiasis. II. Prepatency of *Schistosoma mansoni* in *Biomphalaria glabrata* at diurnally fluctuating temperatures. Zeitschrift fur Parasitenkunde (Berlin, Germany) 66: 221-229.

24. Shoukry NM, el-Assal FM, Soluman GN, Mansour NS (1997) Susceptibility of three successive snail generations from positive and negative laboratory bred *Biomphalaria alexandrina* from different localities in Egypt to infection with *Schistosoma mansoni* from Giza. J Egypt Soc Parasitol 27: 317-329.

25. Cridland C (1968) Results of exposure of batches from highly susceptible and less-susceptible strains of *Biomphalaria alexandrina alexandrina* from Egypt to strains of *Schistosoma mansoni* from Cairo and Alexandria. Bull World Health Organ 39: 955.

26. Richards CS (1975) Genetic factors in susceptibility of *Biomphalaria glabrata* for different strains of *Schistosoma mansoni*. Parasitology 70: 231-241.

27. Cridland CC (1970) Susceptibility of the small *Biomphalaria alexandrina alexandrina* from the UAR and the Sudan to infection with a strain of *Schistosoma mansoni* from Tanzania. Bull World Health Organ 43: 809-815.

28. Files VS (1951) A study of the vector-parasite relationships in *Schistosoma mansoni*. Parasitology 41: 264-269.

29. Fried B, LaTerra R, Kim Y (2002) Emergence of cercariae of *Echinostoma caproni* and *Schistosoma mansoni* from *Biomphalaria glabrata* under different laboratory conditions. J Helminthol 76: 369-371.

30. Kazibwe F, Makanga B, Rubaire-Akiiki C, Ouma J, Kariuki C, et al. (2010) Transmission studies of intestinal schistosomiasis in Lake Albert, Uganda and experimental compatibility of local *Biomphalaria spp*. Parasitol Int 59: 49-53.

31. McClelland WJ (1965) The production of cercariae by *Schistosoma mansoni* and *S. haematobium* and methods for estimating the numbers of cercariae in suspension. Bull World Health Organ 33: 270-276.

32. Frandsen F (1979) Studies of the relationship between *Schistosoma* and their intermediate hosts. III. The genus *Biomphalaria* and *Schistosoma mansoni* from Egypt, Kenya, Sudan, Uganda, West Indies (St. Lucia) and Zaire (two different strains: Katanga and Kinshasa). J Helminthol 53: 321-348.

33. Cooper LA, Ramani SK, Martin AE, Richards CS, Lewis FA (1992) *Schistosoma mansoni* infections in neonatal *Biomphalaria glabrata* snails. The Journal of Parasitology 78: 441-446.

34. Loreau M, Baluku B (1987) Growth and demography of populations of *Biomphalaria pfeifferi* (gastropoda, planorbidae) in the laboratory. J Molluscan Stud 53: 171-177.

35. Anderson RM, Mercer JG, Wilson RA, Carter NP (1982) Transmission of *Schistosoma mansoni* from man to snail: experimental studies of miracidial survival and infectivity in relation to larval age, water temperature, host size and host age. Parasitology 85 (Pt 2): 339-360.

36. Lawson JR, Wilson RA (1980) The survival of the cercariae of *Schistosoma mansoni* in relation to water temperature and glycogen utilization. Parasitology 81: 337-348.

37. Ghandour AM, Webbe G (1973) A study of the death of *Schistosoma mansoni* cercariae during penetration of mammalian host skin: the influence of the ages of the cercariae and of the host. Int J Parasitol 3: 789-794.
